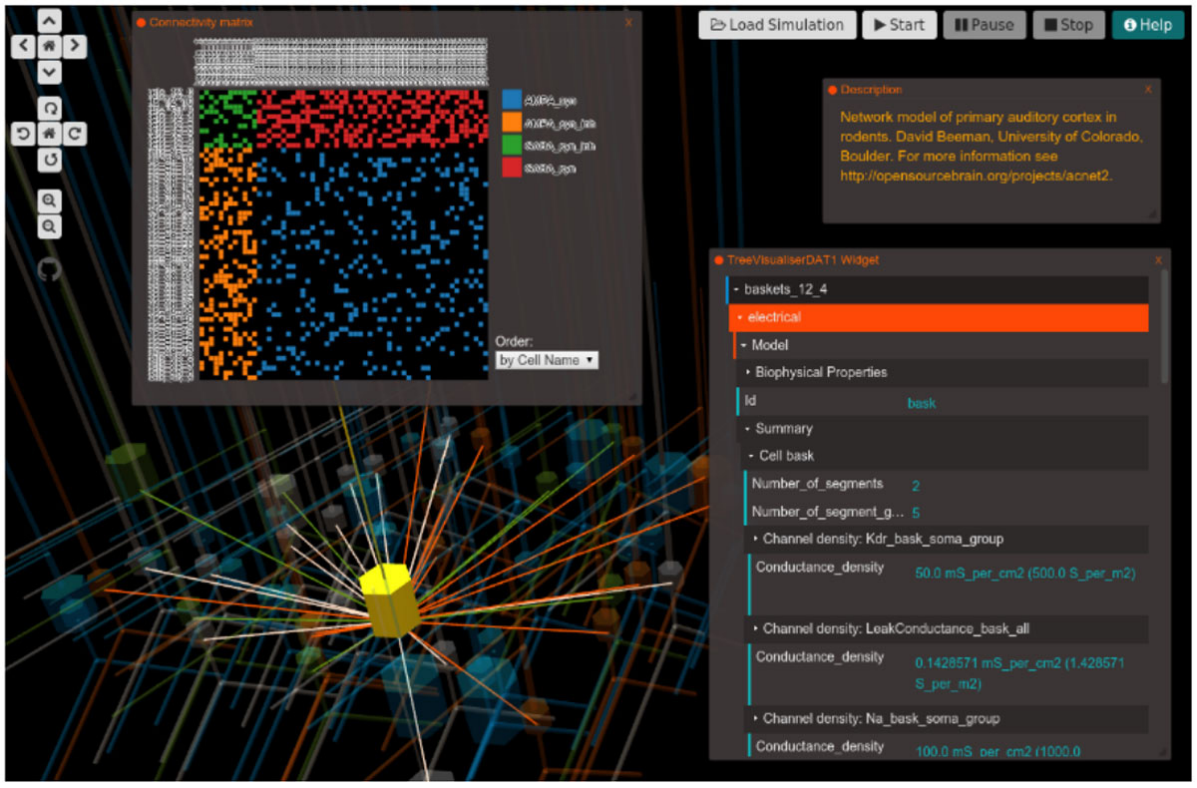# Visualizing, editing and simulating neuronal models with the Open Source Brain 3D explorer

**DOI:** 10.1186/1471-2202-16-S1-P82

**Published:** 2015-12-18

**Authors:** Adrian Quintana, Matteo Cantarelli, Boris Marin, R Angus Silver, Padraig Gleeson

**Affiliations:** 1 Department of Neuroscience, Physiology and Pharmacology, University College London, London, UK; 2 Metacell LLC, San Diego, CA, USA

## 

Reproducibility, accessibility, standardization, and provenance tracking are some of the main challenges for the global scientific community today. In computational neuroscience, making models available in open and accessible formats is an important strategy for improving transparency and reusability of published models. The Open Source Brain initiative (OSB, www.opensourcebrain.org) is an online platform which aims to facilitate sharing and collaborative development of neuronal models. It provides a central location for models of multiple brain regions and species and a set of tools to analyze, edit, visualize and simulate them. While OSB can host models in any format, converting models to NeuroML (specifically v2.0, [1]) is actively encouraged as it allows OSB to access the internal dynamics of the model. NeuroML2 is a simulator independent model description language for computational neuroscience. It has been built on LEMS (Low Entropy Model Specification, [1]), a general purpose language developed to provide a machine readable way of expressing the structure and dynamics of physical models. These languages facilitate cross-simulator validation, which is a crucial factor in ensuring reproducibility and reusability of models.

OSB currently hosts more than 70 projects, has 350 users, and 40 member labs. The vast majority of its features are accessible to non-registered users. If a specific model has been converted to NeuroML2/LEMS the user can make use of a wide range of advanced features in the Open Source Brain 3D explorer. The OSB 3D explorer is a graphical framework for analysis and simulation of neural models based on Geppetto (http://www.geppetto.org). Geppetto, an open source modular platform for complex biological systems, was originally developed as part of the Open Worm project (http://www.openworm.org, [2]). OSB 3D explorer provides a 3D canvas in order to display the model neurons/network. In addition, a wide variety of "widgets" allow the user to access data on both the model and the simulation. A Tree Visualiser widget allows the user to analyze the dynamics of the model, visualize a summary and to modify aspects of the model. The model can be downloaded in different formats (such as NEURON, Brian, Matlab, etc.) or can be simulated on a server/cluster. Such simulations will be executed asynchronously and the user can manage the simulations and analyze the results through a web dashboard. Some other widgets provide tools to analyze the connections in the network, inspect variables during the simulation or generate plots showing for instance the dynamics of a gate or the membrane potential of a cell during the simulation. Every change in the model and simulation outputs is stored in the database providing the user traceability and reproducibility of the whole experiment.

**Figure 1:**